# Adult-Onset Still’s Disease: A Case Report and Review of Current Therapeutic Options

**DOI:** 10.7759/cureus.22743

**Published:** 2022-03-01

**Authors:** Steffi Thomas, Vartika Kesarwani, Melanie Graber, Weishali Joshi

**Affiliations:** 1 Department of Rheumatology, University of Connecticut, Farmington, USA; 2 Department of Internal Medicine, University of Connecticut, Farmington, USA; 3 Department of Rheumatology, University of Connecticut Health, Farmington, USA; 4 Department of Rheumatology, Saint Francis Hospital and Medical Center, Hartford, USA

**Keywords:** treatment, still’s disease, interleukin-6 inhibitors, tocilizumab, adult-onset still’s disease

## Abstract

Adult-onset Still’s disease (AOSD) is a rare autoinflammatory disease that typically presents with a triad of fever, evanescent rash, and arthritis. There is often a delay in diagnosis of AOSD due to its nonspecific clinical presentation, which may mimic other infectious, rheumatological disorders, and malignancies. Corticosteroids have been the cornerstone for the management of AOSD for the past many years. However, with the expanding understanding of its pathogenesis, novel therapeutic options targeting various cytokines are being increasingly recognized. Herein, we present a case of AOSD that was successfully treated with tocilizumab, a monoclonal antibody against the interleukin-6 (IL-6) receptor. For the purpose of this article, we also conducted a literature search to review the current therapeutic options available for the treatment of AOSD.

## Introduction

Adult-onset Still’s disease (AOSD) is a rare autoinflammatory disease with an unclear etiology. AOSD was first described in children by George Still in 1896 [1-4]. The term AOSD was first used in 1971 by Eric Bywaters to describe a series of adult patients presenting with symptoms similar to systemic juvenile idiopathic arthritis [2]. Although the pathogenesis of AOSD is not fully understood, it has been suggested that both infectious and genetic factors play a role [5]. It typically presents with a triad of fever, evanescent rash, and arthritis. The clinical presentation of AOSD is non-specific and can mimic other infectious, rheumatological, autoinflammatory diseases, and hematological malignancies. Therefore, AOSD is a diagnosis of exclusion [5]. Corticosteroids are used as the first line treatment of AOSD, however, newer treatment modalities, including tumor necrosis factor-alpha (TNF-alpha) and interleukin inhibitors, are increasingly being used. Herein, we present a case of AOSD who was successfully treated with tocilizumab (TCZ). We also provide a brief review of the currently available therapeutic options for AOSD.

## Case presentation

A 32-year-old Nepalese female with no known past medical history presented to the hospital with a two-week history of fever and arthralgias. Two weeks prior to the presentation, she developed a sore throat for which she was seen in the emergency department. She was diagnosed with viral upper respiratory illness and treated with over-the-counter antihistamines. Her symptoms failed to improve, and she subsequently developed fever and polyarthralgia. She reported daily morning spikes of fever with a reported highest temperature of 103° Fahrenheit. The review of systems was positive for headaches, myalgia, fatigue, difficulty swallowing, and an evanescent rash. 

Her vital signs on presentation were stable. Her musculoskeletal examination was significant for tenderness at the metacarpophalangeal, proximal, and distal interphalangeal joints of bilateral hands, bilateral wrists, bilateral knees, bilateral ankles, metatarsal, and proximal and distal interphalangeal joints of the bilateral feet. There was also swelling of the bilateral wrists and knees. The skin examination was significant for multiple erythematous, non-blanchable macules on the anterior aspect of the chest and left lateral aspect of the neck. The remainder of the physical examination, including cardiopulmonary, abdominal, and neurological examination, was unremarkable. 

Initial laboratory studies showed an elevated white blood cell count of 18.7 K/uL with 83.1% neutrophils, 11.5% lymphocytes, hemoglobin 10.1 g/dL, hematocrit 31.2%, and platelets 469 K/uL. The workup for anemia revealed elevated serum ferritin of 6905 ng/mL. Infectious workup, including blood cultures, lumbar puncture, hepatitis panel, human immunodeficiency virus (HIV), and tick-borne diseases, was unremarkable. A chest X-ray, computed tomography of the abdomen and pelvis, and ultrasound of the abdomen failed to identify the etiology of her fever. Her inflammatory markers, including erythrocyte sedimentation rate (ESR) and C-reactive protein (CRP), were elevated at 75 mm/hr and 21.6 mg/dL, respectively. Workup for an autoimmune etiology, including antinuclear antibody (ANA), perinuclear antineutrophil cytoplasmic antibody (p-ANCA), antineutrophil cytoplasmic antibody (c-ANCA), myeloperoxidase antibody (MPO), proteinase-3 antibody (PR-3), rheumatoid factor (RF), anti-Smith antibody, anti-ribonucleoprotein antibody, and anti-Ro, anti-La, anti-Scl-70, anti-Jo-1, and antimitochondrial antibodies was unremarkable.

Given her clinical presentation with daily fevers, sore throat, polyarthralgia, and workup significant for elevated serum ferritin in the absence of another identifiable cause, including infectious, autoimmune, and hematological etiology, she met the Yamaguchi criteria and was diagnosed with adult-onset Still’s disease. She was treated with high-dose intravenous corticosteroids (1 mg/kg daily) and non-steroidal anti-inflammatory drugs (NSAIDs) with subsequent improvement in her clinical symptoms and inflammatory markers. She was discharged on oral steroids and NSAIDs.

During the follow-up period, attempts at steroid taper led to recurrence of her symptoms and elevation of inflammatory markers requiring multiple re-hospitalizations and treatment with high-dose intravenous steroids (1 mg/kg daily). Given her steroid dependence, a steroid-sparing agent - anakinra (100 mg subcutaneously daily) was added, and steroids were successfully tapered down to <10 mg/day. Three weeks after initiation of anakinra, the patient developed a pruritic rash on her neck, chest, abdomen, and legs. She was initially treated with oral antifungal agents with further worsening of her rash. She was evaluated by dermatology and underwent a skin biopsy that revealed epidermal acanthosis, dyskeratosis, and superficial perivascular dermatitis with eosinophils, suggestive of drug-induced hypersensitivity to anakinra. Anakinra was discontinued, with subsequent resolution of the rash. The dose of her steroids was increased, and she was started on tocilizumab 4 mg/kg every eight weeks and methotrexate 10 mg weekly. The steroids were successfully tapered off over the next few months. Her symptoms completely resolved, and her inflammatory markers normalized with tocilizumab and methotrexate. After eight months of treatment, she continued to be in remission as her symptoms resolved and inflammatory markers normalized. Tocilizumab was subsequently discontinued. She was continued on methotrexate, which is now being tapered down as the patient continues to be in remission.

## Discussion

AOSD is a rare, chronic, systemic inflammatory disease involving both the innate and adaptive immune systems [5]. It is characterized by quotidian fever, arthritis, and evanescent rash. The term AOSD was first used in 1971 to describe a series of adult patients presenting with symptoms similar to systemic juvenile idiopathic arthritis [2]. Due to the rarity of the disease, it is difficult to estimate the true incidence and epidemiological characteristics. A recent 10-year retrospective study of AOSD in the French population placed the incidence at 0.16 per 100,000 inhabitants [6]. This study also demonstrated that AOSD affects males and females equally and follows a bimodal age distribution pattern, with one peak occurring between the ages of 15 and 25 and the second between the ages of 36 and 46 [6].

The clinical presentation of AOSD is non-specific and can mimic other infectious, rheumatological, autoinflammatory diseases and hematological malignancies. The characteristic clinical manifestations of the disease include spiking quotidian or double-quotidian fever, evanescent salmon-colored macular or maculopapular eruptions, and mild transient oligo-articular arthritis. Rare manifestations like liver disease, ranging from mild transaminitis and hepatomegaly to fulminant liver failure, and cardiopulmonary pathology, including pericarditis, pleural effusions, pulmonary infiltrates, and life-threatening cardiac arrhythmias have also been described. Macrophage activation syndrome (MAS) is a rare but fatal complication found in approximately 1.7% of the patients with AOSD [7]. The clinical course of AOSD can follow three main patterns: monophasic, intermittent, and chronic.

The diagnosis of AOSD is a diagnosis of exclusion and is based on the presence of characteristic clinical and laboratory features. A striking elevation in serum ferritin is the characteristic laboratory finding seen in about 70% of the patients with AOSD and has been shown to correlate with the disease activity [8-11]. Other common laboratory findings include leukocytosis with white blood cell count >15,000 cells/microL, anemia with hemoglobin <10 g/dL, and reactive thrombocytosis. Various classification criteria have been developed for the diagnosis of AOSD, but their use is limited for research purposes due to the lack of sensitivity and specificity required for clinical diagnosis [12-16]. The Yamaguchi criteria (Table 1) are the most widely used classification criteria that consist of four major and five minor criteria [13].

**Table 1:** Yamaguchi criteria for the diagnosis of AOSD The Yamaguchi criteria require the presence of five features, with at least two being major diagnostic criteria. AOSD: adult-onset Still’s disease

Major criteria:	
	Fever of at least 39ºC (102.2ºF) lasting at least one week.
	Arthralgias or arthritis lasting two weeks or longer.
	A nonpruritic macular or maculopapular skin rash that is salmon-colored in appearance and usually found over the trunk or extremities during febrile episodes.
	Leukocytosis (10,000/microL or greater), with at least 80 percent granulocytes.
	Minor criteria:	Sore throat.
Lymphadenopathy.	
Hepatomegaly or splenomegaly.	
Abnormal liver function studies, particularly elevations in aspartate and alanine aminotransferase and lactate dehydrogenase concentrations.	
Negative tests for antinuclear antibody (ANA) and rheumatoid factor (RF).	

Due to the rarity of the condition, there are no guidelines for the management of AOSD. The treatment is consensus-based, and the choice of therapy depends on the severity of the disease and the presence or absence of MAS at the time of presentation. Mild to moderate disease is usually managed with NSAIDs alone and glucocorticoids are only added in cases of inadequate response. However, moderate to severe disease and the presence of MAS require treatment with high-dose glucocorticoids and biologics. The pathophysiology and underlying cause of this disease are poorly understood. High circulating levels of various cytokines, including interleukin (IL)-1, IL-6, IL-18, tumor necrosis factor-α (TNF-α), and interferon-γ (IFN-γ) play a central role in the pathogenesis of the disease and therefore serve as potential targets for therapeutic agents.

NSAIDs and glucocorticoids are the first line therapies used in the treatment of AOSD. Disease-modifying anti-rheumatic drugs (DMARDs) have been used with some degree of benefit; for example, methotrexate, azathioprine, leflunomide, and cyclosporin have been tried in the past although with very limited success [1,17-18]. Treatment becomes challenging in patients who respond poorly to high doses of steroids and those in combination with other DMARDs, as they would relapse during the tapering of the steroids [1,19]. Approximately 17%-32% of AOSD patients fail to respond [20].

Biologics agents, such as TNF-alpha inhibitors including etanercept [21-22], infliximab [22-25], and adalimumab [21,26] are also being used in patients who fail or achieve inadequate response with glucocorticoids and DMARDs. These agents have been predominantly beneficial in treating the arthritic features and show a variable response for fever and cutaneous manifestations. However, they are less effective as compared to other cytokine-targeting agents such as IL-1 and IL-6 inhibitors.

Recently, major progress has been made in the treatment of autoinflammatory disease, especially with the approval of IL-1 and IL-6 inhibitors. Most of these drugs have been mainly investigated in controlled clinical trials in patients with sJIA. However, since AOSD shares the same pathogenic mechanism as systemic juvenile idiopathic arthritis (sJIA) and is believed to represent a continuum of sJIA, the drugs that are approved for sJIA have also shown clinical benefit in AOSD [27-33]. This has been demonstrated by several case series and small retrospective studies, however, many clinical trials to study the effectiveness of these drugs in AOSD are lacking.

Anakinra and canakinumab are the most widely used IL-1 antagonists. Canakinumab has been approved by the Food and Drug Administration (FDA) for use in AOSD [21,29,34-35]. These agents have been shown to be effective both as monotherapy for moderate to severe disease and as a combination with glucocorticoids for AOSD with MAS [29-30]. There are no studies comparing the effectiveness of anakinra with canakinumab, and the choice of agent is usually based on the side-effect profile, duration of therapy, and provider preference. Canakinumab is longer-acting, requiring monthly injections as opposed to daily injections of anakinra and, therefore, it is preferred for chronic management. Canakinumab is usually added when the patients fail to respond to other agents including anakinra [34].

Rilonacept, also known as IL-1 trap, is an IL-1a and b inhibitor that has been shown to be effective in treating both the systemic and arthritic manifestations of refractory AOSD. It is mostly used in patients who have not had much response to anakinra [36]. In a study done by Henderson et al., five refractory AOSD patients were treated with rilonacept, and three of five patients were noted to have an improvement in their symptoms.

Tocilizumab (TCZ), a humanized monoclonal antibody against the IL-6 receptor, has been shown to be effective for the treatment of systemic and arthritic features of AOSD. High levels of IL-6 have been associated with an arthritic presentation in both sJIA and AOSD and, therefore, it has been proposed that tocilizumab may be superior to IL-1 blocking agents in patients with predominant arthritic symptoms [37-38]. A study by de Voysson et al. found that 30 out of 35 patients (86%) treated with TCZ achieved a prompt improvement in articular manifestation and 27/28 (96%) achieved improvement in systemic symptoms [1,39]. About 80% of the patients achieved a decrease in their steroid therapy and 20% of them were able to discontinue them. Furthermore, Kaneko et al. conducted a randomized double-blind placebo-controlled study of 27 patients and found that TCZ was effective in improving the patients’ symptoms at four weeks and decreasing the glucocorticoid dosages. Although in this study, the p-value was 0.24 and did not achieve statistical significance, 61.5% of the patients in the TCZ group and 30.8% (95% CI 9.1 to 61.4) in the placebo group achieved the primary outcome of ACR50 (American College of Rheumatology) - response at four weeks. Based on this study, TCZ is currently approved for use in AOSD in Japan for patients who have not responded to standard therapies.

In addition to the above study, Wang et al. conducted an observational study that included AOSD patients refractory to steroids and other traditional DMARDs found 28 of them had significant improvement in their symptoms and inflammatory markers at eight weeks when TCZ (intravenous 8 mg/kg) was added to methotrexate (oral 12.5 mg once per week) [1,39]. This response was sustained even after 48 weeks into treatment. The study also noted a decrease in the dosage of oral prednisone after 48 weeks of treatment. Sarilumab, another IL-6 receptor inhibitor, has only been used once per Ma et al. [36].

Interferon-gamma appears to play a key pathogenic role in MAS and therefore JAK inhibitors like tofacitinib have been shown to be effective for the treatment of AOSD with MAS [40]. It has been used in China in 14 patients with refractory AOSD. Seven of the 14 patients achieved complete remission and six of 14 achieved partial remission and one relapsed with lowering of the prednisone dose [36,40]. Baracitinib has also been used in one case report in a corticosteroid-dependent refractory AOSD in 2019 [36,41].

The use of other biologic agents, such as abatacept and rituximab, has also been reported. Abatacept, a CTLA4 inhibitor, has also shown some success in refractory AOSD patients who were unresponsive to DMARDS, anakinra, and adalimumab. Rituximab, a chimeric anti-CD20 monoclonal antibody, has also been used in refractory AOSD.

There have also been a couple of investigational drugs undergoing research as a treatment modality. Tadekinig alfa, a recombinant IL-18 binding protein, is an investigational agent that has shown preliminary evidence of efficacy in some patients with AOSD [42]. It is not yet commercially available. Emapalumab, an anti-IFN-gamma autobody, is under investigation as a potential treatment of AOSD with MAS.

Our patient, who was initially treated with high-dose steroids and unfortunately had an allergic skin reaction with anakinra, was treated successfully with the combination of TCZ and methotrexate. She is currently off TCZ for the past five months and is in the process of tapering methotrexate.

## Conclusions

Tocilizumab has shown to be effective in managing patients with AOSD refractory to traditional and conventional treatments with promising data. Our patient, who was initially treated with high-dose steroids and, unfortunately, had an allergic skin reaction with anakinra, was treated successfully with the combination of TCZ and methotrexate. She is currently off TCZ for the past five months and is in the process of tapering methotrexate off. However, TCZ is not currently approved by the FDA as one of the treatment options for AOSD. Therefore, a multicenter collaboration study needs to be conducted to provide a better profile, visibility, and validation of TCZ as a treatment option for patients with AOSD.
